# Ferroptosis: New Dawn for Overcoming the Cardio-Cerebrovascular Diseases

**DOI:** 10.3389/fcell.2021.733908

**Published:** 2021-11-11

**Authors:** Meng-Yi Luo, Jian-Hui Su, Shao-Xin Gong, Na Liang, Wen-Qian Huang, Wei Chen, Ai-Ping Wang, Ying Tian

**Affiliations:** ^1^ Institute of Clinical Research, Affiliated Nanhua Hospital, University of South China, Hengyang, China; ^2^ Hengyang Key Laboratory of Neurodegeneration and Cognitive Impairment, Department of Physiology, Institute of Neuroscience Research, Hengyang Medical College, University of South China, Hengyang, China; ^3^ Department of Pathology, First Affiliated Hospital, University of South China, Hengyang, China; ^4^ Department of Anesthesiology, Affiliated Nanhua Hospital, University of South China, Hengyang, China

**Keywords:** ferroptosis, cardio-cerebrovascular disease, inflammation, iron, lipid peroxidation

## Abstract

The dynamic balance of cardiomyocytes and neurons is essential to maintain the normal physiological functions of heart and brain. If excessive cells die in tissues, serious Cardio-Cerebrovascular Diseases would occur, namely, hypertension, myocardial infarction, and ischemic stroke. The regulation of cell death plays a role in promoting or alleviating Cardio-Cerebrovascular Diseases. Ferroptosis is an iron-dependent new type of cell death that has been proved to occur in a variety of diseases. In our review, we focus on the critical role of ferroptosis and its regulatory mechanisms involved in Cardio-Cerebrovascular Diseases, and discuss the important function of ferroptosis-related inhibitors in order to propose potential implications for the prevention and treatment of Cardio-Cerebrovascular Diseases.

## 1 Introduction

Cardio-Cerebrovascular Diseases (CCVDs) are the collective terms for cardiovascular and cerebrovascular diseases, which refer to ischemic or hemorrhagic diseases of heart, brain and systemic tissues caused by hyperlipidemia, hyperglycemia and hypertension (Vasan et al., 2001; McEvoy 2012), including atherosclerosis (AS), myocardial infarction (MI), heart failure (HF), diabetic cardiomyopathy (DCM), hypertension, ischemic stroke (IS), and so on. According to epidemiological surveys, CCVDs are very common among middle-aged people over 50, and have become the top one killer of people (Dalen et al., 2014). Patients with CCVDs are gradually becoming younger (Singh et al., 2015), accounting for almost one-third of global deaths every year (2015). CCVDs are characterized by high prevalence, disability and mortality rates (Zhang et al., 2011). Even with the application of the most advanced and perfect treatments available, more than 50% of survivors with cardio-cerebrovascular accidents may still be unable to take care of themselves completely (DeVon et al., 2017). However, the existing methods are not ideal for the prevention and treatment of CCVDs, and there is a lack of effective therapeutic strategies to improve neurological function. Therefore, in order to prevent and treat the disease and improve prognosis, there is an urgent need to explore the pathogenesis of CCVDs, as well as new therapeutic strategies. During these years, the role of cell death has been emerging in CCVDs (Lu et al., 2021).

Cell death (CD) is essential for maintaining homeostasis and basic biological functions within tissues, and its changes are of great importance in disease pathology. Different types of CD have their own manifestations, and many types of CD are defined by differences in morphological and biochemical characteristics (Galluzzi et al., 2012). Apoptosis is one of the first types of CD to be recognized, and is essential for maintaining homeostasis in the cardio-cerebrovascular internal environment (Favaloro et al., 2012). Both decreased and increased apoptosis can lead to pathological changes. The maintenance of normal structure and function of the cardio-cerebrovascular system requires a balance between the production and death of cells (including cardiomyocytes, endothelial cells, vascular smooth muscle cells, and neurons) in the tissues and organs (Zhaolin et al., 2019). Excess CD often leads to dysfunction. Consequently, we began to ponder over whether there are any other ways of death (i.e., necroptosis, pyroptosis, and ferroptosis) that play an important role in CCVDs as apoptosis?

Based on the functional differences, CD can be divided into accidental cell death (ACD) and regulated cell death (RCD). RCD concerns signal cascade reactions involving effector molecules, and has unique biochemical characteristics, morphological features and immunological consequences. For the past few years, many studies have explored the role of a new type of RCD-ferroptosis in CCVDs (Gao et al., 2015; Tuo et al., 2017). Ferroptosis, first proposed by Stockwell’s group in 2012, is an iron-dependent modality of RCD and characterized by lipid peroxide accumulation, which leads to oxidative damage to cell membranes, and differs from other forms of RCD in several ways (Dixon et al., 2012). Necroptosis is a type of RCD with necrotic features including cell membrane rupture and organelle swelling, and finally would cause inflammatory response. The activation of mixed lineage kinase domain-like protein (MLKL) by receptor-interacting protein 3 (RIPK3) is a key regulatory pathway of necroptosis, which disrupts cell membrane integrity and causes inflammation. Pyroptosis is a kind of RCD activated by inflammasomes, which is accompanied by cell swelling, cell membrane lysis and the release of inflammatory factors. Autophagy refers to a process that the concave membrane structure wraps senescent organelles and substances and then forms autophagosomes. Under the action of acidic lysosomal enzymes, the wrapped substances are degraded to achieve intracellular environmental homeostasis and organelle renewal. Autophagy plays an important role in neuro-toxicity and neuronal death induced by hypoxia-ischemia. The four RCD types differ largely in morphology, biochemistry and genetics. Ferroptosis is a way to regulate CD caused by iron overload and reactive oxygen species (ROS)-dependent lipid peroxides accumulation, with structural changes focused on mitochondria (Shojaie et al., 2020). Necroptosis and pyroptosis are characterized by the formation of plasma membrane pores via caspase-1, which leads to the pro-inflammatory cytokines release and cell lysis, and thus controls the inflammatory response. It is a profound inflammatory pattern of RCD associated with the innate immune system (Orning et al., 2018). Autophagy is an intracellular waste degradation pathway that is activated in response to cellular stress and usually mediates protective rather than cytotoxic effects (Galluzzi et al., 2018). Except for the differences, what remains the same is that they are all tightly regulated by intracellular signaling pathways. However, ferroptosis plays a key role in the pathogenesis of several CCVDs, and studies have shown that ferroptosis is closely related to AS, MI, HF, DCM, hypertension, and IS (Wu et al., 2021a). More importantly, in many cases, inhibiting ferroptosis through different mechanisms has been proved to have a protective effect on CCVDs (Magtanong and Dixon 2018; Fang et al., 2019). Thus, ferroptosis is a potential therapeutic target. In summary, the discovery of ferroptosis expands our understanding of CD in CCVDs, and targeting ferroptosis will provide a new strategy and broaden thoughts for the prevention and treatment of CCVDs.

## 2 Contribution of Ferroptosis in Cardio-Cerebrovascular Disease

As the maintenance of cardio-cerebrovascular homeostasis depends on CD and cell renewal, excessive cell loss will contribute to the occurrence of many CCVDs (Lu et al., 2020). Hence, ferroptosis may participate in the occurrence, development and prognosis of diseases through the damage to normal tissues and organs or the loss of specific functions.

Studies have stated that heart tissue is easy to accumulate free iron, and high dietary iron intake increases the risk of CCVDs. Specific inhibitors of ferroptosis have been proved to effectively reduce heart damage and have cardio-protective effects (Fang et al., 2020). Cell damage in the infarct region of the brain is an inevitable upshot of focal cerebral ischemia (Masaldan et al., 2019; Datta et al., 2020), and delaying CD by targeting ferroptosis can save dysfunctional neurons and reduce the size of infarct region. Accordingly, inhibiting ferroptosis can effectively prevent and alleviate the progression of CCVDs.

### 2.1 The Role of Ferroptosis in Atherosclerosis

**FIGURE 1:**
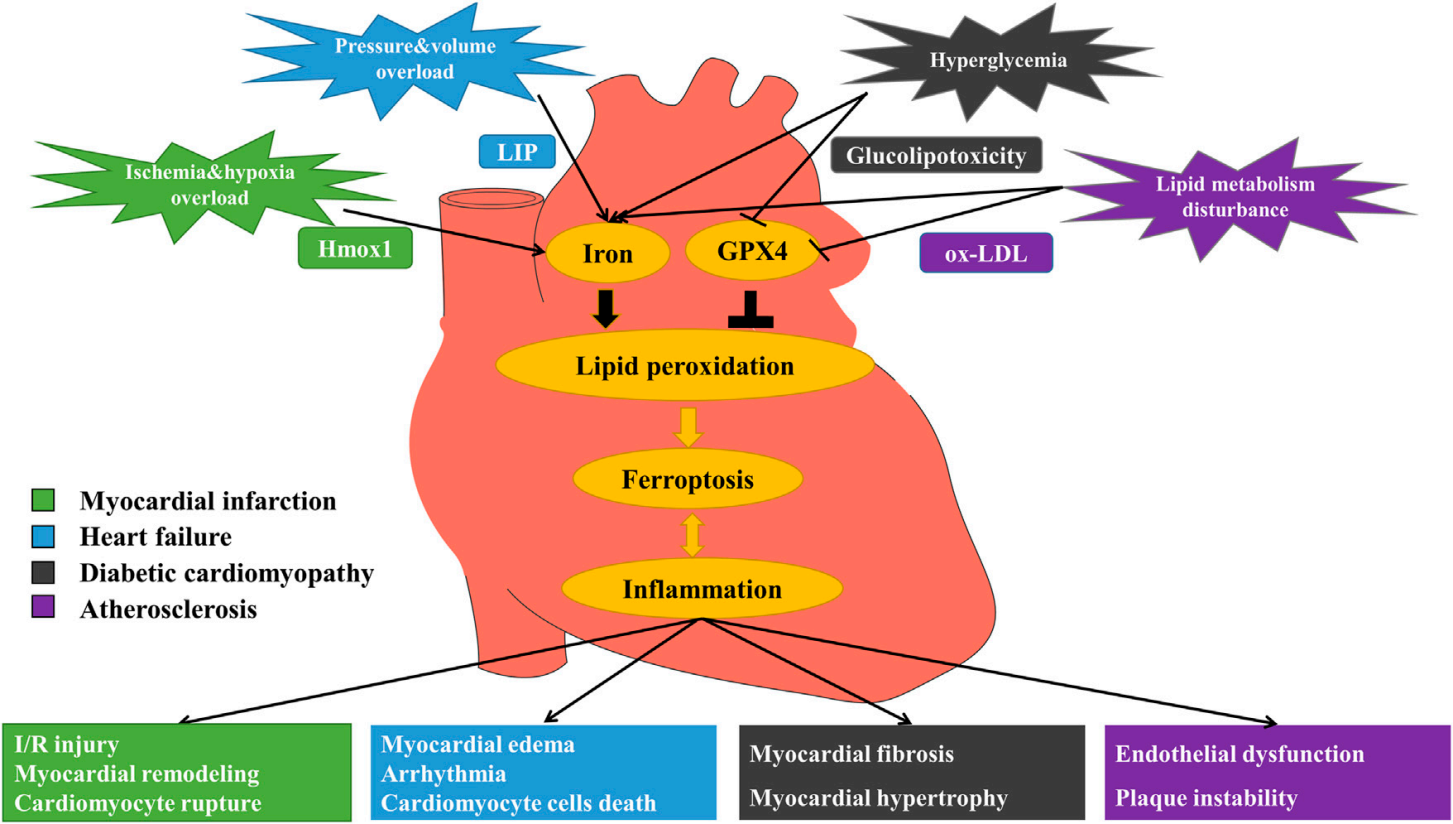
Mechanism of ferroptosis in myocardial infarction (MI), heart failure (HF), diabetic cardiomyopathy (DCM) and atherosclerosis (AS). MI: Under the stimulation of ischemia and hypoxia, excessive free iron releases via up-regulation of heme oxygenase 1 (Hmox1), promoting ferroptosis and eventually causing I/R injury, myocardial remodeling and myocardial cell rupture. HF: Stimulated by pressure and volume overload, labile iron pool (LIP) formation increases, leading to iron overload and the occurrence of ferroptosis, and then causing myocardial edema, arrhythmia and cardiomyocyte cell death. DCM: In the circumstance of hyperglycemia, free iron increases and glucolipotoxicity induces ferroptosis, triggering myocardial fibrosis, and myocardial hypertrophy. AS: When there exists lipid metabolism disturbance, iron overload and oxidized low-density lipoprotein (ox-LDL) induce lipid peroxidation that triggers ferroptosis, finally causing endothelial dysfunction and plaque instability.

AS is a chronic progressive vascular disease characterized by disturbances in lipid metabolism (Weber and Noels 2011) and narrowing of the arterial lumen through the formation of atherosclerotic plaques in the arterial wall (Veseli et al., 2017; Moore and Tabas 2011). Endothelial dysfunction and inflammation are closely associated with the development of AS (Gimbrone and García-Cardeña 2016; Li et al., 2021b). Lipid peroxidation has been shown to be associated with endothelial dysfunction and inflammatory responses, and plays a key role in the pathogenesis of AS (Hammad et al., 2009; Gimbrone and García-Cardeña 2016). Firstly, in human and animal models, iron levels and inflammatory mediators in atherosclerotic lesions were significantly elevated compared to healthy arterial tissues (Ross 1999; Sullivan 2009). On the one hand, iron overload led to endothelial dysfunction by enhancing the oxidative and inflammatory responses in endothelial cells (ECs) (Gimbrone and García-Cardeña 2016; Xu 2019). On the other hand, iron overload induced ferroptosis in foam cells which led to plaque instability, and ferroptosis in foam cells increased IL-1 and IL-8 expression, promoting inflammatory responses (Su et al., 2021). Secondly, in ApoE^-/-^ AS mice fed with high-fat diet, inhibition of ferroptosis by Ferrostatin-1 (Fer-1) protected against thoracic aortic lipid peroxidation and inhibited exacerbation of AS (Bai et al., 2020). In addition, inhibition of ferroptosis inhibited lipid peroxidation induced by oxidized low density lipoprotein (ox-LDL) and endothelial dysfunction in mouse aortic endothelial cells (MAECs) *in vitro*. Fer-1 also down-regulated the expression of adhesion molecules and up-regulated eNOS expression (Bai et al., 2020) (Figure 1). Similarly, lowering iron levels in atherosclerotic tissue with the iron chelator Deferoxamine (DFO) could inhibit ferroptosis and inflammation, and mitigate the development of AS (Zhang et al., 2010). Finally, other studies found that serum PDSS2 and nuclear factor erythroid-2-related factor 2 (Nrf2) levels were significantly reduced in AS patients (Yang et al., 2021). PDSS2 is a key enzyme for the synthesis of coenzyme Q10 (CoQ10) (Li Y. et al., 2018). Over-expression of PDSS2 in human coronary artery endothelial cells (HCAECs) inhibited ROS release and iron content to suppress ferroptosis and promote the proliferation of HCAECs. PDSS2 also attenuated ferroptosis in HCAECs by activating the antioxidant factor NF-E2-related factor 2 (Nrf2) (Yang et al., 2021). Nrf2 is the primary transcription factor that regulates antioxidant responses and inhibits ferroptosis in various cell types by protecting cells from lethal ROS stress (Gorrini et al., 2013; Dodson et al., 2019). MiRNA17-92 (miR-17-92) is a multifunctional oncogenic miRNA cluster that plays an important role in tumor angiogenesis and tissue development (Mendell 2008). Over-expression of miR-17-92 in HUVECs can protect ECs from erastin-induced ferroptosis by targeting the zinc lipoprotein (A20)-acyl-CoA synthetase long-chain family member 4 (ACSL4) axis that could protect glutathione peroxidase 4 (GPX4) and inhibit ROS production (Xiao et al., 2019). Macrophages, as one of the important immune cells in AS, play an vital role in the inflammatory response and foam cell formation (Xie et al., 2020). The current study shows that iron levels in plaque tissue, particularly in macrophages, are significantly elevated and are associated with AS (Thong, Selley, and Watt 1996; Bi et al., 2021). It has not been proved yet whether ferroptosis occurs in macrophages during AS. Due to lipid peroxidation, plaque hemorrhage and iron deposition are markers of progressive plaque in humans, and ferroptosis in cells may persist (Martinet et al., 2019).

In conclusion, ferroptosis is associated with the multiple vascular cytopathic processes that contribute to the development and progression of AS. Ferroptosis promotes the development of AS through inflammation, endothelial cell dysfunction, and the formation of foam cells, using ferroptosis inhibitors in AS could reverse these effects, which may provide new targets and strategies for the prevention and treatment of AS.

### 2.2 The Role of Ferroptosis in Myocardial Infarction

MI refers to severe and persistent myocardial ischemia caused by sharp decrease or interruption of arterial blood flow, resulting in myocardial ischemic necrosis. Refractory MI has gradually become one of the most serious diseases in the world (Zhang, Yu, et al., 2020). In MI, complete coronary artery occlusion is accompanied by a variety of structurally and functionally adverse consequences, the most irreversible of which is myocardial cell death.

Some studies have revealed that inhibiting myocardial cell death can reduce the size of MI (Del Re et al., 2019). It has been proved that Fe^2+^, erastin and RAS-selective lethal 3 (RSL3) can induce cardiomyocyte cell death (Kobayashi et al., 2018); and in the early and middle stages of MI, antioxidant GPX4 is detected at a decreased level (Wang H. et al., 2020). The content of Fe^2+^ is strictly regulated, and when its level is out of balance, excess ferrous will provide electron-promoting lipid peroxidation via Fenton reaction, resulting in an increased production of ROS and inducing ferroptosis (Kajarabille and Latunde-Dada 2019; Anandhan et al., 2020). Erastin and RSL3 are specific inducers of ferroptosis by inhibiting System X_c_^−^ and GPX4, respectively. In the acute myocardial infarction (AMI) model, the expression of divalent metal-ion transport 1 (DMT1) was found to be significantly up-regulated. DMT1, a member of solute carrier family 11 member 2 (SLC11A2), plays a role in iron transport, and its expression would be increased if exposed to high-iron environment. Over-expression of DMT1 can promote hypoxia/reperfusion-induced ferroptosis in cardiomyocyte cells, while its knockdown can suppress CD (Song et al., 2021). Besides, cardiomyocytes depend heavily on mitochondrial respiration to gain ATP as an energy source, thus mitochondrial dysfunction is an important cause of cardiomyocyte cell death (Patel and Karch 2020). In the animal model, iron chelator Deferiprone (DFP) improves myocardial mitochondrial function, and Liproxstatin-1 (Lip-1), a specific inhibitor of ferroptosis, also protects the integrity of mitochondrial structure (Ravingerová et al., 2020; Wu et al., 2021a). The evidence above suggests that ferroptosis is involved in the occurrence of MI, and the inhibition of ferroptosis can reduce the loss of cardiomyocytes and alleviate the progression of MI.

In terms of mechanism, ferroptosis promotes MI mainly through three pathways. During ferroptosis, heme oxygenase 1 (Hmox1) is up-regulated with increased free iron release from cardiomyocytes, and then left ventricular remodeling would be triggered, causing severely impaired function of left ventricular in MI patients (Baba et al., 2018). Besides, oxidative stress (OS) in cardiomyocytes would be increased, aggravating ischemia/reperfusion (I/R) injury (Song et al., 2021) (Figure 1). At the same time, ferroptosis is accompanied by excessive ROS production, resulting in depolarization of the mitochondrial membrane potential and opening of the mitochondrial permeability transition pore (MPTP), further leading to cell rupture (Ravingerová et al., 2020). Also, ferroptosis contributes to the accentuated myocardial remodeling and more severe dysfunction after MI by triggering inflammatory responses (Huang and Frangogiannis 2018). Therefore, blocking ferroptosis-related targets is expected to treat MI.

### 2.3 The Role of Ferroptosis in Heart Failure

HF is not an independent disease, but the final stage of the development in most primary Cardiovascular Diseases, contributing to high morbidity and mortality worldwide and a huge social and economic burden on the healthcare system (Wu et al., 2021b). Keeping the dynamic balance of cardiomyocytes plays a key role in maintaining the normal physiological function of heart (Ma et al., 2020), and the loss of cardiomyocytes caused by CD would induce and aggravate the development of HF.

According to related reports on HF, myocardial hemorrhage can cause the increase of labile iron pool (LIP) production and trigger the release of free iron into myocardium, being one of the important pathogenesis of HF (Qiu et al., 2020). LIP is a pool of weakly chelated iron with redox activity and is an ionic source available for Fenton reaction (Jamnongkan et al., 2017). Higher than normal levels of LIP, can disrupt the balance of redox and consequently promote ferroptosis in cardiomyocytes (Lou et al., 2021). There is a study has found that the contents of LIP and lipid peroxides were increased in the rat model of HF induced by pressure overload, suggesting that HF is associated with the lipid peroxidation process which is highly related to iron-dependent metabolism. In addition, ferroptosis in cardiomyocytes also occurred in doxorubicin (DOX)-induced cardiotoxic mice model. In the HF model, it showed that GPX4 and ferritin heavy chain1 (FTH1) expression levels were down-regulated (Chen et al., 2019). GPX4 is considered to be the key enzyme of ferroptosis and its genetic inactivation or pharmacological inhibition would cause the accumulation of lipid peroxidation products, which is a crucial hallmark of ferroptosis (Stockwell et al., 2020). FTH1 is a ferroptosis-related protein that is reduced in cells where ferroptosis occurs. Previous studies have demonstrated that inhibition of mitochondrial death pathway could protect cardiac tissues from HF (Abdelwahid et al., 2017), while the opening of mitochondrial voltage-dependent anion channel (VDAC)2/3 induced by erastin could increase the uptake of labile iron and the generation of ROS, leading to ferroptosis in cells (Wang Y. et al., 2020). Iron chelator Deferasirox (DFX) has been proved to reduce the iron content of cardiac tissue in HF, thereby playing a role in cardiac protection. Besides, other specific inhibitors of ferroptosis also have the ability to alleviate disease progression in various clinical models, such as heart disease models (Fang et al., 2020). Hence, it can be determined that ferroptosis increases the risk of HF by accelerating the damage and loss of cardiomyocytes.

As an important cause of cardiomyocyte cell death during HF, the mechanisms of ferroptosis are mainly through the following four aspects. To start with, in the process of ferroptosis, Hmox1 is activated, which promotes the increase of LIP and destroys cell homeostatic capacity, thus compromising the integrity of cardiomyocytes (Kakhlon and Cabantchik 2002). Secondly, since cardiomyocytes are easily affected by free iron overload, the release of excess free iron during ferroptosis would result in up-regulation of transferrin receptor (TFR) and down-regulation of ferritin, promoting cardiomyocyte cell death through mitochondrial VDAC2/3 or membrane lipid peroxidation through Fenton reaction to induce myocardial edema and dysfunction (Liu et al., 2018). Afterwards, excessive ROS would interfere with Ca^2+^ homeostasis in cardiomyocytes, affecting multiple ion transporters responsible for myocardial electrical activity and bringing about diastolic and systolic dysfunction and arrhythmia. Lastly, ferroptosis can trigger systemic inflammatory response, activate multiple pro-inflammatory signaling, induce cardiomyocyte hypertrophy, fibrosis and death, and eventually lead to chronic adverse ventricular remodeling (Shirazi et al., 2017; Huang and Frangogiannis 2018) (Figure 1). The level of pro-inflammatory cytokines is closely related to the severity and poor prognosis of chronic HF (Thackeray et al., 2018). To sum up, one of the key pathogenic factors of lethal HF is the irreversible dysfunction and death of terminally differentiated cardiomyocytes (Fang et al., 2019). Inhibition of ferroptosis has notable clinical significance to prevent cardiomyocyte inflammation, hypertrophy and death, and to maintain normal cardiac function.

### 2.4 The Role of Ferroptosis in Diabetic Cardiomyopathy

Diabetes is a pro-inflammatory disease (De Blasio et al., 2020). CCVDs are the leading cause of death in patients with diabetes. DCM is a chronic and irreversible cardiac complication (Dillmann 2019), and is difficult to prevent from progressing to significant HF (Zhang J. et al., 2020). Therefore, there is a need to find new therapeutic approaches to treat DCM more effectively. The pathogenesis of DCM is multifactorial and complex, including hyperglycemia, fatty acids, OS, inflammation, endoplasmic reticulum stress (ERS), myocardial fibrosis and hypertrophy (Bugger and Abel 2014). Many of the molecular mechanisms that increase fibrosis and myocardial inflammation can activate ferroptosis signaling pathway. Fatty acid oxidation or nicotinamide adenine dinucleotide phosphate (NADPH) oxidase in diabetes produces elevated levels of ROS, leading to myocardial death, inflammation and fibrosis that impair cardiac structure and function (Parim et al., 2019). OS due to an imbalance in ROS production and antioxidant capacity has been suggested as a common mechanism leading to inflammation and DCM (Liu et al., 2014; Lv et al., 2019). Lipid peroxide accumulation caused by ROS and fatty acid oxidation is a major cause of ferroptosis (Cao and Dixon 2016), leading to myocardial death, inflammation, fibrosis, and damage to cardiac structure and function (Li W. et al., 2017). Elevated ROS and OS are common features of inflammation (Lv et al., 2019), altering inflammatory phenotype in DCM, including increased expression of cell adhesion molecules, increased macrophage and leukocyte infiltration, and increased expression of inflammatory cytokines (Westermann et al., 2007; Singh et al., 2008; Rajesh et al., 2012). Biopsy showed that the apoptosis of a diabetic heart was 85 times higher than that of the nondiabetic heart, indicating that cardiomyocytes in diabetes were sensitive to suffer CD (e.g. ferroptosis) (Cai and Kang 2003). Essentially, CD is the end of cardiomyocytes during DCM (Chen et al., 2020a). It has been shown that in a DCM mouse model, glucolipotoxicity effects resulting from chronic diabetes, would promote lipid peroxidation; while inactivating Nrf2-mediated antioxidant defenses and impairing Nrf2-coordinated iron metabolism (Chien et al., 2020) would promote the expression of ferroptosis and exacerbate the progression of DCM (Zang et al., 2020). More and more research suggests that effective treatment of DCM requires a specific endogenous antioxidant defense system, rather than non-selective scavenging of ROS that may impair physiological redox signaling (Chen et al., 2014). Many natural and synthetic Nrf2 activators ameliorate OS and iron metabolism disorders, reduce lipid peroxidation and inflammation, showing good therapeutic effects in animal models with DCM (Hu et al., 2018; Wang et al., 2018). These findings all indicate that ferroptosis promotes the development of DCM through abnormal iron metabolism, lipid peroxidation, ROS production, and enhanced cardiomyocyte inflammation (Figure 1). Therefore, regulation of ferroptosis in cardiomyocyte and attenuation of myocardial inflammation in DCM by Nrf2 may provide new therapeutic options for DCM.

### 2.5 The Role of Ferroptosis in Hypertension

**FIGURE 2:**
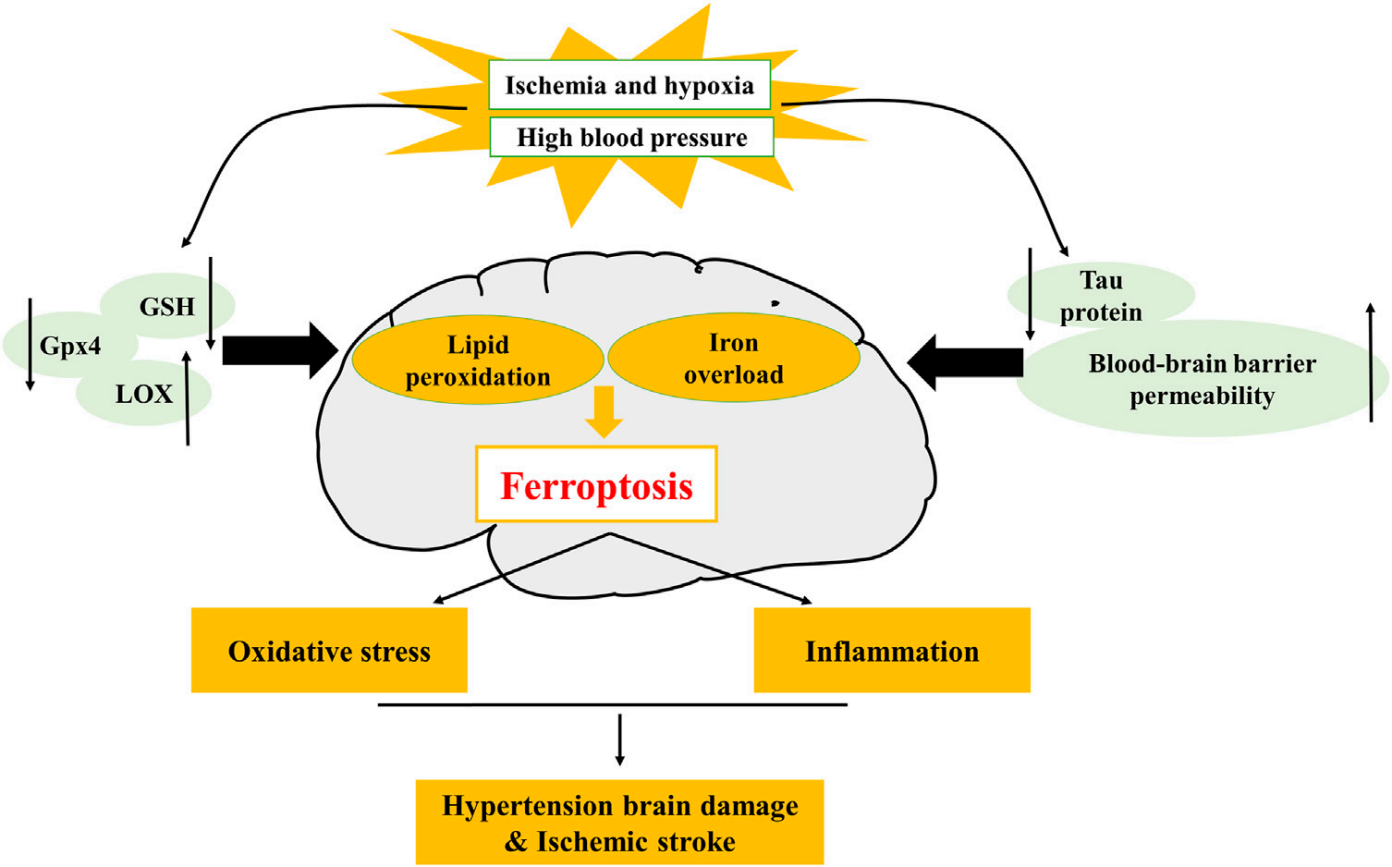
Mechanism of ferroptosis in hypertension and ischemic stroke. The decrease of glutathione peroxidase 4 (GPX4), glutathione (GSH), tau protein, and the increase of lipoxygenase (LOX), blood-brain barrier (BBB) permeability, can lead to the occurrence of ferroptosis. LOX is involved in hypertensive brain injury, tau protein and BBB permeability are involved in ischemic stroke.

Hypertension is one of the major risk factors of CCVDs (Zhang Y. et al., 2020). As blood pressure rises, the incidence of CCVDs increases (Pistoia et al., 2016). Brain is an early target from organ damage due to elevated changes in blood pressure (Sierra et al., 2011), and hypertensive brain injury is a serious complication of hypertension. Brain tissue is particularly vulnerable to OS and highly susceptible to oxygen radical damage because of its high oxygen consumption and lack of antioxidant enzymes. In addition, brain tissue is rich in unsaturated fatty acids, which are targets of lipid peroxidation (Dringen 2000; Nunomura et al., 2012; Zhang et al., 2018). Over these years, studies have demonstrated that OS mediates pathological changes in the brain and blood vessels (Dumitrescu et al., 2018), and ferroptosis is caused by OS and lipid peroxidation (Kerins and Ooi 2018; Morris et al., 2018) that are closely associated with brain injury and neuro-degenerative diseases. Studies have confirmed that ferroptosis is linked to a variety of central nervous system disorders, such as Parkinson's syndrome, epilepsy and stroke (Stockwell et al., 2017). Brain tissues in hypertensive brain-injured rats exhibited changes in ferroptosis-relevant indicators, such as decreased expression of GPX4, glutathione (GSH), increased iron content and malondialdehyde (MDA). This shows that hypertension leads to iron overload in the brain, and iron overload increases OS and lipid peroxidation, thereby causing ferroptosis (Yang et al., 2020) in neurons and ultimately leading to brain injury (Figure 2). However, how does hypertension lead to iron overload in the brain and what are specific mechanisms of ferroptosis in hypertensive brain injury are unknown. Therefore, whether inhibition of ferroptosis can play a protective role against hypertensive brain injury remains to be further investigated.

### 2.6 The Role of Ferroptosis in Ischemic Stroke

IS refers to hemiplegia and disturbance of consciousness caused by cerebral infarction and clogged cerebral arteries, and is becoming a major global issue affecting human health, causing death and long-lasting disability. When the blood flows into a certain portion of brain and then is obstructed, IS would occur. This process is followed by hypoxia and nutritional deprivation, which leads to neuronal excitotoxicity and death (Shen et al., 2020). CD in the infarct region is the most inevitable consequence of focal cerebral ischemia (Datta et al., 2020). It was found that after cerebral injury, there were iron accumulation, metabolic disorder, increased expression of ferroptosis-related genes, GPX4 inactivation, ROS and pro-inflammatory factors generation (Li J. et al., 2020). The specific manifestations were iron overload, decreased GSH level and the enhancement of lipid peroxidation. Thusly, ferroptosis is considered to be an important pathway leading to IS.

It has been proved that iron deposition was increased in the basal ganglia, thalami, periventricular and subcortical white matter areas after severe ischemic and hypoxic brain injury (Li J. et al., 2020). Following iron accumulation, the neuronal damage during reperfusion would be aggravated (Tuo et al., 2017). Moreover, cerebral ischemia decreased the level of tau protein (Lu et al., 2021). Tau protein has the ability of facilitating neuronal iron efflux (Tuo et al., 2017). If tau protein is pathologically reduced, it would cause iron ion transport disorder, thereby leading to ferroptosis in cells. Also, when blood-brain barrier (BBB) is disrupted by stroke and other brain diseases, it helps to transfer the systemic iron pool into neurons in the brain parenchyma, exacerbating ferroptosis (Datta et al., 2020; DeGregorio-Rocasolano et al., 2019). Additionally, iron chelators have been shown to improve the prognosis of IS in mammals (Wu et al., 2018). It was found that the administration of ferroptosis inhibitors partially protected neurons from death in the middle cerebral artery occlusion (MCAO) model (Weiland et al., 2019). Similarly, an increase in lipid peroxidation levels and a decrease in GSH levels could also be detected in the same above model (Li J. et al., 2020). GSH is the most abundant small-molecule antioxidant in cells (Bayır et al., 2020). As an effective substrate of GPX4 and an indispensable cofactor in GPX4 activation, GSH maintains GPX4 activity indirectly to inhibit the occurrence of ferroptosis. Selenocysteine peptides can transport selenium to the ventricle and improve the functional recovery after IS (Alim et al., 2019). Selenium is able to maintain the activity of GPX4 (Chen et al., 2020b) and improve neurological functions. Inactivation of GPX4 leads to neuronal death both *in vivo* and *in vitro* (Kenny et al., 2019). Cui et al. found that ACSL4 gene knockout could attenuate ischemic brain injury while over-expression aggravated IS (Cui et al., 2021). ACSL4 is not only a marker for ferroptosis sensitivity, but also an indispensable down-stream player in the ferroptosis process (Doll and Conrad, 2017; Seibt et al., 2019). Based on studies concerning ferroptosis and IS, there is growing body of evidence that ferroptosis would be induced during IS and then aggravate brain damage, suggesting that ferroptosis may be a potential target for preventing and treating IS.

Currently, it has been reported that multiple regulatory mechanisms of ferroptosis mediate neuronal death, including iron overload, OS and neuro-inflammation. During IS, tau protein decreases, generous free iron deposits, and then Fe^2+^ releases electrons, producing excessive ROS. At the same time, iron-dependent lipoxygenase (LOX) catalyzes polyunsaturated fatty acids (PUFAs) to induce OS, which further alters the permeability of BBB (Iadecola and Anrather 2011). At this point, the injured brain tissue rapidly produces pro-inflammatory cytokines, causing leukocyte infiltration and eventually inducing ferroptosis and exaggerating brain edema, neuron death and progress of IS (Jin et al., 2013) (Figure 2). Generally, iron accumulation induces OS, bringing about the increase of ROS levels and the imbalance of antioxidant balance and infiltrating pro-inflammatory mediators that further amplify cerebral inflammatory responses, which aggravate neuronal damage during reperfusion (Jin et al., 2013; Tuo et al., 2017). Ferroptosis inhibitor or iron chelation therapy can significantly lessen cerebral I/R injury and ameliorate the prognosis of patients with stroke. Targeting or combined with ferroptosis pathway to regulate the cerebral environment is a very advisable treatment at present, and the inhibition of ferroptosis is likely to provide a new strategy for the prevention of neuron death induced by IS.

### 2.7 The Role of Ferroptosis in Other Cerebrovascular Diseases

#### 2.7.1 The Role of Ferroptosis in Intracerebral Hemorrhage

Intracerebral hemorrhage (ICH) refers to bleeding caused by non-traumatic vascular rupture in the brain parenchyma. Its incidence is relatively low, but only a part of patients could survive for more than 1 month, and most of them suffer from disability (Feigin et al., 2009; van Asch et al., 2010), which contribute to the high mortality of ICH. A large amount of studies have shown that the blood accumulates and flows into the surrounding brain and then compresses the tissue during ICH, which may be followed by multiple forms of CD, resulting collectively in neuronal death; at the same time, since the intracerebral hematoma releases hemoglobin (Hb)-based neurotoxin (Wang 2010), the neural damages secondary to perihematomal edema (PHE) that is caused by BBB disruption also occur in the brain parenchyma, becoming an important cause of morbidity and mortality in patients (Jeon et al., 2021).

The release of Hb is due to the accumulation and dissolution of iron-rich erythrocytes in the brain parenchyma, which consequently leads to iron toxicity and induces the production of ROS (Wu et al., 2011), interfering with normal function of cells and resulting in neuronal death (Zecca et al., 2004). Li et al. (Li et al., 2018) demonstrated for the first time that ferroptosis occurred in the collagenase-induced ICH mouse model, and ferroptosis was observed in neurons within the 1mm range of the edge from hematoma by transmission electron microscopy. Consistently, Zille et al. (Zille et al., 2017) also found that Hb could induce ferroptosis in primary cortical neurons. Ferroptosis is characterized by iron-dependent ROS accumulation and lipid peroxidation. The normal functions of antioxidant GPX4 and cystine transporter System X_c_^−^ are of great importance in controlling ferroptosis; while iron chelators, lipid peroxidation inhibitors and antioxidants can effectively inhibit the occurrence of ferroptosis. Iron deposition is a common indicator of ICH injury. Speer et al. (Speer et al., 2013) found that iron chelators were able to suppress ferroptosis by regulating hypoxia-inducible factor-1 (HIF-1) , thus playing a neuro-protective role in ICH. Chang et al. (Chang et al., 2014) showed that epicatechin could affect iron metabolism via regulating ferroptosis-related genes such as IREB2, protecting mice from ICH. In addition, GPX4 levels decreased in rat ICH models, while Fer-1, belonging to lipid peroxidation inhibitors, could block ferroptosis and improve secondary brain injury (Zhang et al., 2018). Similarly, mice treated with Lip-1 after ICH showed a more mild degree of neurologic deficits and less lesion volume (Li et al., 2017). N-acetyl cysteine (NAC) is an antioxidant that enhances GSH synthesis, increases cysteine level and System X_c_^−^ activity, thereby inhibiting ferroptosis in Hb-induced ICH model and improving the prognosis of ICH mice (Karuppagounder et al., 2018).

Based on the above evidence, we can draw the view that ferroptosis is related to the neuronal death after ICH; inhibition of ferroptosis can effectively attenuate secondary injury after ICH and play a neuro-protective role.

#### 2.7.2 The Role of Ferroptosis in Subarachnoid Hemorrhage

Subarachnoid hemorrhage (SAH) is a common acute cerebrovascular event in clinic, and its survivors will experience severe neurological disability. As a devastating subtype of stroke, the morbidity and mortality rates of SAH are pretty high (Li Y. et al., 2021). In recent years, the pathophysiological mechanisms of early brain injury (EBI) after SAH have become the hotspot of experimental and clinical research, and EBI is the key factor of poor prognosis of SAH. EBI is a complex and multifactorial process involving reduced cerebral blood flow, BBB disruption, lipid peroxidation and other pathological events. These pathological events exacerbate the injury, and finally lead to neuronal death which in turn leads to neurological dysfunction (Yan et al., 2015).

There are many modalities of CD in EBI. Li et al. (Li Y. et al., 2021) found that inhibiting ferroptosis could ameliorate CD in the brain and neuronal death in the cell model by improving BBB permeability and cerebral edema, thus alleviating EBI after SAH. ACSL4 is able to induce ferroptosis, and increasing its expression would exacerbate inflammation, OS and cognitive deficits after SAH (Qu et al., 2021); however, over-expression of GPX4, could reduce the brain water content and improve neurological behavior by suppressing ferroptosis (Gao et al., 2020). Similarly, in *in vivo* experiments, Lip-1, a ferroptosis inhibitor, decreased neuronal death and neuro-inflammation in EBI by protecting mitochondrial functions and reducing lipid peroxidation, thereby ameliorating cerebral edema and neurological deficits after SAH (Cao et al., 2021). To sum up, ferroptosis is involved in EBI, and inhibition of ferroptosis can attenuate the degree of EBI and have a neuro-protective effect after SAH (Dangol et al., 2019).

## 3 The Mechanism of Ferroptosis

**FIGURE 3:**
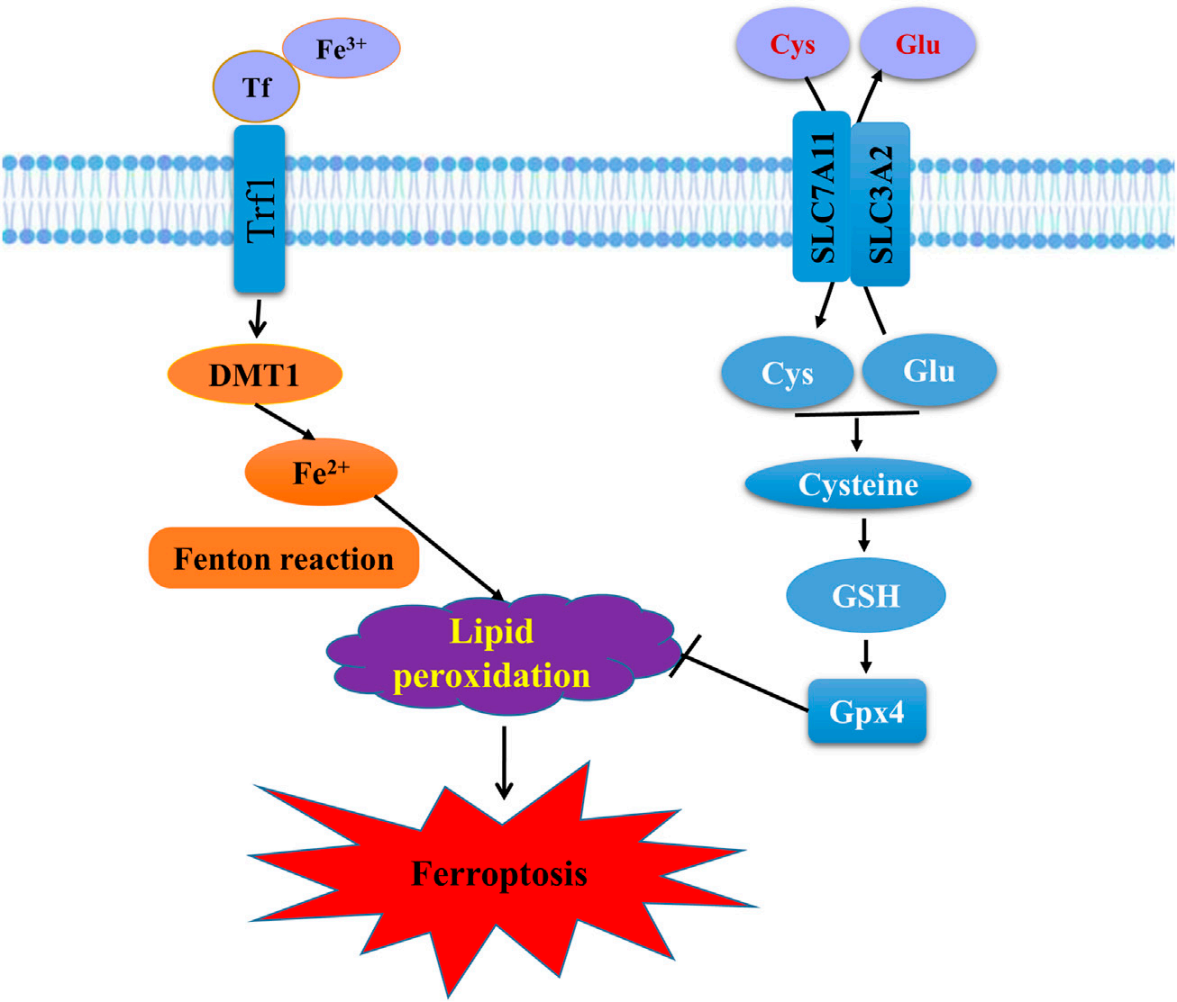
Schematic representation of the mechanism of ferroptosis. Ferroptosis is an iron-dependent form of regulated cell death mediated by iron overload and lipid peroxidation of cellular membranes. Fe^3+^ imported through the transferrin receptor is converted to Fe^2+^ in endosomes and released from endosome by divalent metal-ion transporter 1 (DMT1). Fenton reaction converts Fe^2+^ into Fe^3+^, which induces lipid peroxidation. GPX4 is the major endogenous mechanism to suppress lipid peroxidation. High extracellular concentrations of glutamate (Glu) inhibit system X_c_^−^, which imports cystine (Cys) by exchanging intracellular Glu for extracellular Cys. Cys is subsequently converted to cysteine, which generates GSH, a cofactor for GPX4.

Studies have indicated that ferroptosis may be induced by different physiological conditions and pathological stress. Among these factors, the dynamic balance of iron and the abnormality of lipid peroxidation are the most important to induce ferroptosis, while the functional state of System X_c_^−^ and the activity level of GPX4 are the key pathways to regulate ferroptosis. The relevant mechanisms and implications will be described in detail below (Figure 3).

### 3.1 Inducing Factors of Ferroptosis

#### 3.1.1 Iron (Fe^2+^) Metabolism

As an indispensable cofactor in many enzyme metabolism processes and a catalyst of redox-cycling reaction (Stoyanovsky et al., 2019), iron is involved in a variety of important physiological and biochemical processes *in vivo*. Studies have demonstrated that when iron accumulates in the body, it may be accompanied by OS, inflammation and CD (Masaldan et al., 2019). Since the homeostasis of iron in cells depends on the dynamic balance between its absorption, output and utilization, and thusly, the transport, metabolism and storage of iron should be strictly regulated. Excess iron would result in an increased production of ROS and induce ferroptosis (Kajarabille and Latunde-Dada 2019; Anandhan et al., 2020).

Over-expression of nuclear receptor coactivator 4 (NCOA4) or ATG protein can accelerate the degradation of ferritin, thus increase the level of free iron and aggravate the subsequent oxidative damage during ferroptosis (Liu J. et al., 2020). Traditional Chinese herbal extracts, such as artemisinin, also interfere with iron metabolism, increase Fe^2+^ concentration, and induce ferroptosis (Han et al., 2020). In contrast, Nrf2 is a stress-induced transcription factor responsible for maintaining cell metabolism, redox and protein balance, and controlling the expression of genes that counteract oxidative and electrophilic stress (Dodson et al., 2019). Once Nrf2 is activated, the storage of iron would increase, OS would be inhibited, and the occurrence of ferroptosis could be directly blocked.

#### 3.1.2 Lipid Peroxidation

Lipid peroxidation refers to the oxidation and degradation of lipids. In this process, free radicals “steal” electrons from lipids in the cell membrane, thereby leading to cell damage. In living cells, the most common initiator of lipid peroxidation is ROS. The lethal accumulation of ROS is a sign of ferroptosis (Feng and Stockwell 2018; Hirschhorn and Stockwell 2019; Kajarabille and Latunde-Dada 2019). When the content of ROS is too high and completely destroys the redox dynamic balance, lipid peroxidation will be induced by non-enzymatic and enzymatic pathways, and then result in ferroptosis (Su et al., 2019). Therefore, the abundance and location of intracellular oxidizable substrates also determine the degree of lipid peroxidation and ferroptosis (Han et al., 2020).

Lipoxygenases and phosphorylase kinase G2 are two key drivers of lipid peroxidation (Yang et al., 2016), which can directly damage phospholipids and intrigue ferroptosis. FINO2 is an organic substance containing internal peroxides (Li J. et al., 2020). It promotes widespread lipid peroxidation by directly inducing iron oxide and indirectly inhibiting GPX4, and preferentially initiates ferroptosis (Gaschler et al., 2018; Zhang X. et al., 2020). Hmox-1 can also accelerate erastin-induced ferroptosis by generating ROS. In addition, artemisinin increases ROS levels, and FIN56 binds and activates squalene synthase to accumulate ROS (Kang et al., 2019), leading to the depletion of endogenous antioxidant CoQ10 and then inducing ferroptosis by lipid peroxidation. However, ferroptosis suppressor protein 1 (FSP1) has an inhibitory effect on ferroptosis by reducing CoQ10, inhibiting lipid peroxidation and ferroptosis terminal driver pLPO.

P53, as a gene encoding transcription factor that controls the initiation of cell cycle, has dual regulatory effects on lipid peroxidation. On the one side, p53 promotes the expression of SAT1 (Qiu et al., 2020), and then increased SAT1 cooperates with ROS to lead to lipid peroxidation (Murphy 2016), further oxidizes PUFAs, and finally causes ferroptosis; on the other side, p53 directly inhibits the activity of DPP4 or induces the expression of CDKN1A/p21, to make it difficult for NOX1 to interact with DPP4 to form complexes, thus alleviating lipid peroxidation and ferroptosis (Tao et al., 2020).

### 3.2 The Up-Stream Regulatory Mechanisms of Ferroptosis

#### 3.2.1 System X_c_^−^

The cystine-glutamate antiporter System X_c_^−^ is a disulfide-linked heterodimer composed of a light chain XCT and a heavy chain 4F2, namely, substrate-specific subunit SLC7A11 and regulatory subunit SLC3A2 (Song et al., 2018; Kajarabille and Latunde-Dada 2019; Qiu et al., 2020), with the ability to maintain cell reduction environment. System X_c_^−^, the most up-stream node in the ferroptosis signaling cascade (Sato et al., 2018; Seibt et al., 2019; Stockwell et al., 2020), synthesizes antioxidant GSH by up-taking cystine and exchanging intracellular glutamate at a molar ratio of 1:1 and then reducing cystine to cysteine (Seibt et al., 2019; Stockwell et al., 2020). The administration of System X_c_^−^ inhibitors can deplete intracellular cysteine, reduce GSH concentration, trigger OS, and increase cell sensitivity to ferroptosis (Han et al., 2020).

Small molecule erastin can directly and selectively target and inhibit the System X_c_^−^, and irreversibly bind to and inactivate XCT, leading to continuous disorder of cystine uptake, a decrease in cellular antioxidant capacity, and eventually ROS accumulation and ferroptosis (Sato et al., 2018; Kajarabille and Latunde-Dada 2019; Su et al., 2019). Similarly, BECN1 directly blocks the activity of System X_c_^−^ and initiates ferroptosis by binding to SLC7A11, the core component of System X_c_^−^. P53 down-regulates the expression of SLC7A11 to reduce cystine uptake and enhance the cell sensitivity to ferroptosis.

As an important antioxidant defense system *in vivo*, pharmacological inhibition on System X_c_^−^ can promote the occurrence of lipid peroxidation and ferroptosis. Enhancing the stability of SLC7A11 can inhibit ferroptosis, while the destabilization of SLC7A11 can increase the sensitivity to ferroptosis (Liu et al., 2019). It has been proved to be a feasible treatment strategy that inhibiting the occurrence and development of ferroptosis by regulating the activity of System X_c_^−^ or the expression level of SLC7A11.

#### 3.2.2 Glutathione Peroxidase 4

GPX4 is a selenoprotein enzyme, and its activity is the cornerstone of antioxidant defense (Ursini and Maiorino 2020). GPX4 can interrupt the lipid peroxidation chain reaction by reducing complex hydroperoxides to the corresponding counterparts, to quench lipid peroxidation (Seibt st al., 2019).

RAS-selective lethal 3 (RSL3) is the first described GPX4 inhibitor (Seibt et al., 2019). RSL3 binds to the nucleophilic active site of GPX4, and then reduces the expression level of GPX4 most effectively through chloroacetamide moiety (Jelinek et al., 2018), thus inactivating GPX4, producing lethal ROS accumulation and inducing ferroptosis. ACSL4 is a fatty acid activating enzyme expressed in the outer membrane of mitochondria and endoplasmic reticulum. Its over-expression reduces the activity of GPX4, resulting in increased release of lactic dehydrogenase, decreased cell viability and increased ferroptosis markers. Erastin indirectly blocks the function of GPX4 by depleting GSH, then intrigues ferroptosis. FIN56 promotes the degradation of GPX4 (Belavgeni et al., 2020), that leads to ferroptosis in cells.

Selenium is an essential trace element in mammals. On the one hand, it protects GPX4 from irreversible inactivation; on the other hand, it promotes transcriptional expression of GPX4, protecting cells from OS and inhibiting ferroptosis (Chen et al., 2020b). Nrf2 plays a role in alleviating lipid peroxidation and ferroptosis by regulating the biosynthesis of GSH and promoting the expression of GPX4.

Obviously, ferroptosis is a process regulated by multiple factors (Table 1) and involves many clinical applications. Seeking for valid targets concerning ferroptosis in CCVDs is of great importance.

**TABLE 1:** Initiators and inhibitors of ferroptosis.

Factor	Mechanism	Effect
NCOA4 Liu et al. (2020a)	Promote ferritin degradation	Induction
ATG protein Liu et al. (2020a)	Increase free iron levels	Induction
RAS Han et al. (2020)	Increase iron content, up-regulate TFR and down-regulate ferritin	Induction
LOX Yang et al. (2016)	Cause damage to phospholipids	Induction
Phosphorylase kinase G2		
FINO2 Gaschler et al. (2018)	Induce iron oxide and inhibit GPX4	Induction
Hmox1 Tao et al. (2020)	Supplement intracellular iron and produce ROS	Induction
BECN1 Kang et al. (2018)	Bind to SLC7A11 to block system X_c_^−^ activity	Induction
RSL3 Jelinek et al. (2018)	Bind to GPX4's nucleophilic active site and inactivate GPX4	Induction
ACSL4 Cheng et al. (2020)	Decrease expression of GPX4	Induction
Artemisinin (Traditional Chinese herbal extract) Han et al. (2020)	Interfere with iron metabolism, increase ROS levels and decrease GSH levels	Induction
FIN56 Kang et al. (2019); Belavgeni et al. (2020)	Bind and activate enzyme squalene synthase to cause depletion of CoQ10 and promote GPX4 degradation	Induction
Erastin Sato et al. (2018), Minagawa et al., (2020)	Bind to and inactivate SLC7A11 and deplete the level of GSH	Induction
FSP1(Han et al. 2020)	Reduce CoQ10	Inhibition
Thiazolidinedione (TZDs) (Angeli et al. 2017)	Limit the availability of substrates	Inhibition
Mitochondria-targeted free radical scavenger (MitoQ, MitoTEMPO) Angelova et al. (2021)	Antioxidant effect	Inhibition
Selenium Chen et al. (2020b)	Protect GPX4 and drive GPX4 transcriptional expression	Inhibition
Nrf2 Dodson et al. (2019)	Increase iron storage, prevent OS and regulate GSH biosynthesis	Inhibition
p53 Murphy (2016); Kang et al. (2019)	Increase SAT1 with ROS to increase lipid peroxides and down-regulate expression of SLC7A11	Induction
	Inhibit DPP4 activity or induce CDKN1A/p21 expression to alleviate lipid peroxidation	Inhibition

### 3.3 The Down-Stream Regulatory Mechanisms of Ferroptosis

#### 3.3.1 Ferroptosis and Mitochondrion

Mitochondria are the most abundant organelles in cardiomyocytes, which are mainly responsible for energy metabolism (Li N. et al., 2020); meanwhile, cardiomyocyte as an important source of energy, if its mitochondrial function deteriorates, it would lead to myocardial I/R injury. The double membranes structure of mitochondria provides an ideal location for ferroptosis, and some researchers have observed mitochondrial impairment in patients with ferroptosis (Sha et al., 2021). After ferroptosis, excess intracellular free iron can enter the mitochondria, resulting in smaller myocardial mitochondria, increased mitochondrial membrane density, decreased mitochondrial respiration, and mitochondrial membrane potential depolarization (Abdalkader et al., 2018; Sumneang et al., 2020), thus inducing CD and a series of Cardiac Diseases such as arrhythmia (Hu et al., 2021). A recent study shows that if ferroptosis is suppressed, the production of ROS in mitochondria would be reduced, which can save the toxicity caused by inhibition of mitochondrial complex I (Basit et al., 2017) and effectively prevent cardiac ischemic damage. Ferroptosis inhibitor Lip-1 can reduce the level of VDAC1 and mitochondrial ROS in myocardium, to protect the integrity of mitochondrial structure and exert the myocardial protective effect against I/R injury, reducing the myocardial infarct size (Li N. et al., 2021; Wu et al., 2021a). The above findings suggest that ferroptosis plays a vital role in the pathology of CD through mitochondrial function deteriorates and contributes to the development of Cardiac Diseases.

#### 3.3.2 Ferroptosis and Endoplasmic Reticulum Stress

Ferroptosis can also participate in myocardial I/R injury by modulating ERS (Li N. et al., 2020). On the one hand, it has been proved that the occurrence of ferroptosis is accompanied by the production of ERS (Wang et al., 2019), and ferroptosis can also lead to ERS via cystine-glutamate antiporter System X_c_^−^ (Sun et al., 2015); on the other hand, ERS induces apoptosis through the activation of three up-stream signaling proteins: inositol requiring protein-1, activating transcription factor-6 and protein kinase RNA (PKR)-like ER kinase. Li et al. (Li W. et al., 2020) found that inhibition of ferroptosis could reduce ERS and mitigate myocardial damage in diabetic rats with I/R injury. It is noteworthy that compared with ERS agonist alone, the combination of ferroptosis inhibitor and ERS agonist can significantly reduce the incidence of ERS and cardiomyocyte injury, while ferroptosis agonist can aggravate cardiac dysfunction. Apparently, ferroptosis promotes ERS and cardiomyocytes injury, and aggravates the progression of Cardiac Diseases.

#### 3.3.3 Ferroptosis and Oxidative Stress

As is well known that OS is induced by the imbalance in the redox state. Accumulating evidence suggests that the regulation of ferroptosis can change the level of OS in cardiomyocytes or vascular cells, resulting in cardiomyocyte injury, cardiac hypertrophy and HF through the interaction between ROS with ERS (Kamoshita et al., 2014; Li W. et al., 2020; Li N. et al., 2021).

In summary, CD is pivotal for internal homeostasis and prevention of CCVDs (Duan et al., 2021). Ferroptosis is accompanied by iron overload, excess lipid peroxidation and a large amount of ROS production, which can accelerate a collection of pathological processes such as inflammation, mitochondrial dysfunction, ERS, and OS that further lead to the increase of CD and damage to the normal function of cardio-cerebral vessels.

#### 3.3.4 Ferroptosis and Inflammation

Inflammation is a basic pathological process including infection and tissue damage, and appears when the organism is stimulated by some specific physiological or pathological factors. Different from other types of RCD, ferroptosis is immunogenic (Sun et al., 2020).

It has been reported that when ferroptosis occurs in neurons, then microglia are activated and release toxic substances including pro-inflammatory factor IL-6, which can cause neuro-inflammation and lead to further brain damage (Tang et al., 2020). In addition, ferroptosis also changes the inflammatory phenotype in macrophages (Ouyang et al., 2021), which promotes macrophage polarization and then aggravates inflammation (Yu et al., 2021). Ferroptosis is capable of directly releasing the pro-inflammatory damaged-associated molecular patterns (DAMPs) that promote the development of inflammation and numerous inflammatory diseases. Similarly, after the death of cells that have experienced ferroptosis, intracellular components are also released in the form of DAMPs upon plasma membrane rupture, triggering the immune system to recruit neutrophils to the injured myocardium (Hu et al., 2021). Once there exists an accumulation of DAMPs, it would trigger the amplification of tissue inflammation, leading to more severe inflammation and CD, forming a vicious circle and disrupting the normal physiological function of tissues and organs (Linkermann et al., 2014; Land et al., 2016). Ferroptosis inhibitor Fer-1 can not only inhibit the infiltration of macrophages (Li N. et al., 2020), but also inhibit the adhesion of neutrophils to coronary vascular endothelial cells (Li N. et al., 2021), effectively reducing inflammation and dysfunction in heart injury.

In a word, as a crucial defensive response *in vivo*, inflammation is closely associated with CD induced by ferroptosis. As mentioned previously, inflammation can induce myocardial hypertrophy and fibrosis during cardiac injury, destroy BBB and lead to neurological defects in the ischemic region, promoting the progress of CCVDs. To conclude, iron metabolism and lipid peroxidation contribute to the occurrence of ferroptosis, and eventually up-regulate inflammation. Thus, it can be said that inflammation is an important intermediate process that can be regulated by ferroptosis. In the end, ferroptosis-induced inflammation would trigger or even aggravate development of CCVDs.

## 4 Advances in Drug Application to Improve Cardio-Cerebrovascular by Interfering Ferroptosis

At present, the World Health Organization is committed to the global prevention, management and monitoring of CCVDs, to identify potential mechanisms and effective therapeutic drugs, in order to reduce the incidence, prevalence and mortality of CCVDs (Zhang X. et al., 2020). As one of the results of the imbalance of lipid peroxidation and antioxidant system, ferroptosis has high clinical value in treatment of CCVDs.

For the prevention and control of ferroptosis, we focus on the two main targets in the process of its occurrence and development: iron metabolism and lipid peroxidation. We will concentrate on the drugs or inhibitors acting on the above targets to provide the possibility for the treatment of clinically related CCVDs (Table 2).

**TABLE 2:** Treatment strategy of ferroptosis in cardio-cerebrovascular diseases.

Drug/Inhibitor		Mechanism
Iron chelators	DFO Crisponi, Nurchi, and Lachowicz (2019)	Protect the coordinated Fe^3+^ cation from reduction, avoiding ROS generation via Fenton reaction; bind Fe in the bloodstream and promote its elimination through urine.
	DFP Jansová and Šimůnek (2019)	Have access into myocytes and chelate Fe from the bloodstream and from cells.
	DFX Jansová and Šimůnek (2019)	Bind circulating and intracellular Fe and remove cardiac Fe, attenuating myocardial oxidative stress.
Radical-trapping antioxidants (RTAs)	Fer-1 Liu et al., 2020b)	Trap chain-carrying radicals to eliminates lipid hydroperoxides, producing the same anti-ferroptosis effect as GPX4.
	Lip-1 Fan et al., (2021)	Inhibit mitochondrial lipid peroxidation, and restore the expression of GSH, GPX4 and FSP1.
	Vitamin E Kajarabille and Latunde-Dada (2019)	Inhibit LOX activity by competing at the substrate-binding site and scavenging hydroxyl group radicals; downsize the membrane oxidation level to curb the ferroptosis motivated by GPX4 depletion.
	Vitamin A Su et al., (2019)	Scavenge peroxyl radicals and block the reaction chain to protect lipid membranes from peroxide damage.
	CoQ10 Jafari et al., (2018)	Capture radical intermediates and regenerate tocopherol and ascorbate to suppress lipid peroxidation.
	Vitamin C Kajarabille and Latunde-Dada (2019)	Scavenge free radicals by forming ascorbyl radicals
	GSH Lewerenz and Maher (2011)	Scavenge excessive ROS, protect mitochondrial membranes from free radicals and reduce peroxides.
	NAC Ezeriņa et al., (2018); Moreira et al., (2007)	Maintain intracellular GSH levels and lower endogenous oxidant levels.
Enzymatic antioxidants	SOD He et al., (2017); Li et al., (2020c)	Remove intracellular ROS and reduce lipid peroxide content, being the first line to against oxygen-derived free radicals.
	GPXs Dächert et al., (2016)	Reduce the accumulation of lipid peroxides.

### 4.1 Iron chelators

Iron chelator has the ability to become a stable complex through strong binding with iron ion, thus preventing too much free iron ion from participating in redox reaction in cells to produce ROS, initiate lipid peroxidation, cause cell oxidative damage and trigger ferroptosis. Iron chelators are often used to halt Fenton reaction and related lipid peroxidation, and have been demonstrated to improve some pathological contexts (Conrad et al., 2020).

Currently, there are three specific iron chelators, namely Deferoxamine (DFO), Deferiprone (DFP) and Deferasirox (DFX) (Jansová and Šimůnek 2019).

### 4.2 Deferoxamine

DFO can be used to treat systemic iron overload and acute iron poisoning (Jansová and Šimůnek 2019). By combining free iron in a complex, DFO protects Fe^3+^ from reduction and avoids the formation of ROS through Fenton reaction (Crisponi et al., 2019). DFO can effectively reduce the pathological deposition of iron in organs and protect the heart against I/R injury *in ex vivo* perfused hearts (Kobayashi et al., 2018).

### 4.3 Deferiprone

DFP is an oral iron chelator with high affinity for iron ions. The lipophilicity and low molecular weight of DFP enable it to cross the cellular membranes and gain access into cardiomyocytes (Crisponi et al., 2019), chelating iron from cells (Jansová and Šimůnek 2019). DFP can improve cardiac contractile function and reduce lipid peroxidation and lactate dehydrogenase release. Two *in vitro* studies have shown that DFP can protect neonatal rat cardiomyocytes from cardiotoxicity induced by doxorubicin (Jansová and Šimůnek 2019).

### 4.3 Deferasirox

DFX was the second oral chelator available for clinic use with high oral efficacy and tolerability. It has a high selectivity for Fe^3+^ (Crisponi et al., 2019). It binds circulating iron and intracellular iron, stabilizing the level of iron in cardiac tissue, also clearing cardiac iron and reducing myocardial oxidative stress in Fe overload *in vitro* (Jansová and Šimůnek 2019). The administration of DFX on cardiomyocytes in the early phase of reperfusion can effectively reduce the severity of reperfusion injury (Ravingerová et al., 2020). A Study has shown that DFX improved the severity of acute myocardial infarction, with comparative effects (Nishizawa et al., 2020). Also, DFX can alleviate the lipid peroxidation and endothelial dysfunction in MAECs, inhibiting the occurrence of ferroptosis, and then improving AS lesion (Bai et al., 2020).

### 4.4 Radical-Trapping Antioxidants

RTAs, reacts with chain-carrying peroxyl radicals to produce non-reactive free radicals (Li and Pratt 2015), which destroys the autoxidation of chain-propagating peroxyl radicals (Kajarabille and Latunde-Dada 2019). It is an inhibitor at the initial stage of free radical formation and a chain breaker at the stage of radical breakage and propagation.

RTAs can be divided into lipophilic and hydrophilic free radical scavengers. Ferrostatin-1 (Fer-1), Liproxstatin-1 (Lip-1), and Coenzyme Q10 (CoQ10) are lipophilic; N-acetyl cysteine (NAC) is hydrophilic.

### 4.5 Ferrostatin-1

Fer-1 acts as a lipophilic free radical scavenger due to its redox cycling activity (Doll and Conrad 2017), and alleviates ferroptosis by inhibiting lipid peroxidation and preventing GPX4 deletion or GSH depletion (Zilka et al., 2017; Liu P. et al., 2020). Zille et al. found that Fer-1 was able to rescue mouse primary cortical neurons from CD induced by hemoglobin chloride and hemoglobin *in vitro* (Weiland et al., 2019). Ahmad et al. found that Fer-1 prevented reperfusion injury in mice (Cui et al., 2021); similarly, Tuo et al. found that Fer-1 also protected neurons against cerebral ischemic injury in mouse models (Wu et al., 2018). Moreover, Fer-1 could alleviate AS lesion in high-fat diet (HFD)-fed mice model and improve the viability of MAECs treated with ox-LDL (Bai et al., 2020).

### 4.6 Liproxstatin-1

Lip-1 is a superior antioxidant for capturing free radicals. Lip-1 not only suppresses mitochondrial lipid peroxidation and reduces ROS generation, but also restores GSH, GPX4, and FSP1 levels (Wu et al., 2021a; Fan et al., 2021). Therefore, Lip-1 inhibited ferroptosis with effect in the mice model induced by GPX4 depletion (Doll and Conrad 2017). In addition, a study found that administration of Lip-1 partially rescued neuronal survival and microglia activation, improving neurological function (Cui et al., 2021). There is a study indicated that Lip-1 preserved the expression of GPX4, decreased the expression of ACSL4 and attenuated neuronal injury via inhibition of ferroptosis (Cao et al., 2021).

### 4.7 Coenzyme Q10

CoQ10 is an endogenous antioxidant, a membrane stabiliser and a cofactor. CoQ10 is able to suppress ferroptosis via inhibition of lipid peroxidation by capturing radical intermediates outside of mitochondria (Stockwell et al., 2020). Numerous studies have investigated that CoQ10 increased energy production by mediating electron transfer in the electron transport chain and stabilized myocardial calcium-dependent ion channels to improve contractility of cardiac muscle (Madmani et al., 2014; Jafari et al., 2018); it prevented low-density lipoproteins (LDL) oxidation to maintain normal cardiovascular and cardiac diastolic function, reducing cardiovascular adverse events (Garrido-Maraver et al., 2014); it also reduced OS to prevent lipid peroxidation on cardiomyocyte membrane and inhibit ferroptosis in cardiomyocytes. In CCVDs, CoQ10 could directly act on ECs to stimulate vasodilation and lower blood pressure; it could reduce left ventricular diastolic dysfunction caused by diabetes; it also slows down the process of ferroptosis in cardiomyocytes during ischemia period to alleviate MI and HF (Zozina et al., 2018); it reduces the release of neurohormonal or diminishes neuro-inflammation to improve the prognosis of IS as well (Yang et al., 2015).

### 4.8 N-Acetyl Cysteine

NAC is a precursor of GSH synthesis and an antioxidant that scavenges free radicals and oxidants (Yesilbursa et al., 2006; Ezeriņa et al., 2018), protecting cells from free radical injury, inflammation and ferroptosis. It is reported that NAC protected neurons under ischemic stimulation by preserving mitochondrial function and reducing OS (Moreira et al., 2007). Maryam et al. found that the administration of NAC early after acute ischemic stroke improved neurological impairment and prognosis (Sabetghadam et al., 2020); and ischemic animal model pretreated with NAC showed a decrease in the volume of cerebral infarction, a decrease in neuronal cell death and an improvement in neurological function (Zhang et al., 2014). NAC may effectively reduce cardiac iron level, exert antioxidant and anti-inflammatory effects, improving cardiac and cognitive functions (Sumneang et al., 2019). Study by Xiao et al. has demonstrated that in the model of HF, NAC enhanced antioxidant capacity *in vivo* and reduce cardiomyocyte cell death (Wu et al., 2014). César et al. found that treatment with NAC after MI effectively reduced arrhythmia, cardiac remodeling and infarction expansion (Costa et al., 2020). Furthermore, studies by Dilek and Azita et al. manifested that NAC significantly alleviated OS and I/R injury in patients with MI, improved left ventricular systolic function and attenuated cardiac remodeling (Yesilbursa et al., 2006; Talasaz et al., 2014).

With the deepening of research, ferroptosis has been found in more and more CCVDs. It is of great theoretical significance and practical value to clarify the mechanism of ferroptosis and its role in various diseases, and to develop corresponding inhibitory drugs according to different stages and targets of ferroptosis. This is also the future direction of ferroptosis research (Li J. et al., 2020).

## 5 Conclusion and Prospect

Globally, CCVDs are the major causes of death and disability. Understanding the pathological process of myocardial and neural cell injury is key to developing protective strategies for CCVDs. Iron overload has been found to play a pathogenic role in cardio-cerebrovascular toxicity both in animal models and in patients with different CCVDs. Ferroptosis, an iron-dependent form of regulated cell death, has received increasing attention and has been associated with the pathology of CCVDs. Many studies have shown that ferroptosis occurs in AS, MI, HF, DCM, hypertensive brain injury, and IS. Inhibitors of ferroptosis may prevent these diseases by inhibiting ferroptosis in cardiac and neuronal cells.

Cardio-cerebrovascular cell death is a fundamental pathological process and ferroptosis occurs during all periods of CCVDs. Although reduced levels of ferroptosis inhibitors (e.g. GSH, GPX4, and Nrf2) and changes in the expression of several other genes are known to occur during CCVDs, current assays are not suitable for routine clinical diagnosis. The molecular mechanisms that lead to ferroptosis in CCVDs are still largely unknown. One of the main features of ferroptosis is impaired mitochondrial structure, but it is not easy to observe changes in mitochondria. In the last few years, an increasing number of targets that induce or inhibit ferroptosis have been uncovered, but which of them provides the best therapeutic effect, which can be used in animal models or patients, and which can be easily handled chemically are still unclear. Although research data on ferroptosis are becoming more detailed and complete, most studies have only revealed the phenomenon of ferroptosis in CCVDs, not its specific mechanisms, location, and stages. More importantly, do other types of RCD have an effect on ferroptosis, and if so, is it synergistic or antagonistic? Moreover, although ferroptosis leads to pathological changes such as inflammation and ERS, in many cases, they actually interact with each other. It is not clear which one is in the absolute up-stream or down-stream. Therefore, it is more difficult to elucidate the complete pathway through which ferroptosis acts. For this reason, we must continue to explore the different roles of ferroptosis in different diseases in order to find efficient and targeted therapeutic approaches. We need to clarify the specific pathways of ferroptosis and figure out whether ferroptosis could act on other cell types. Moreover, when does ferroptosis exert its maximum effect after organism damage? Are there any other factors that could induce ferroptosis except for iron overload and lipid peroxidation? What are the specific effects of different ferroptosis inhibitors? Is combined use of drugs more effective than single use? These are fields that we need to study in the future. Therefore, there is still a long way to go before the findings based on animal or cellular experiments are finally applied in clinical practice.

In summary, ferroptosis is closely associated with the pathogenesis of various CCVDs, and with continued research, inhibition of ferroptosis to prevent cardio-cerebrovascular cell death has the potential to be an effective strategy for the treatment of CCVDs.
